# Comparative Transcriptomics of Olfactory Rosettes Reveals Expression Divergence and Adaptive Evolution in Herbivorous and Carnivorous Xenocyprididae Fishes

**DOI:** 10.3390/ani15182741

**Published:** 2025-09-19

**Authors:** Hua Xue, Hailong Gu, Liu Yang, Jingchen Chen, Wenqiao Tang

**Affiliations:** 1 Shanghai Universities Key Laboratory of Marine Animal Taxonomy and Evolution, Shanghai Ocean University, Shanghai 201306, China; zfysqzr@163.com (H.X.); 18091529293@163.com (L.Y.); cjc20141109@163.com (J.C.); 2 Taizhou Institute of Agricultural Science, Jiangsu Academy of Agricultural Sciences, Taizhou 225300, China; 20142208@jaas.ac.cn; 3 Key Laboratory of Exploration and Utilization of Aquatic Genetic Resources, Ministry of Education, Shanghai Ocean University, Shanghai 201306, China

**Keywords:** Xenocyprididae, olfactory system, RNA-seq, feeding adaptation, single-copy orthologous, positive selection

## Abstract

**Simple Summary:**

Olfaction plays a crucial role in regulating feeding behaviors and ecological adaptation in fish. However, the molecular mechanisms underlying olfactory adaptation in fish species with distinct feeding habits remain largely unexplored. In this study, we conducted comparative transcriptomic analyses of olfactory rosettes from four Xenocyprididae species representing herbivorous and carnivorous diets. We identified and functionally annotated unigenes, screened olfaction-related genes, and analyzed the expression patterns of single-copy orthologs. Furthermore, positive selection analysis revealed molecular evolutionary signals associated with olfactory function. Our findings demonstrate distinct expression trends and evolutionary patterns between herbivorous and carnivorous species, providing new insights into the functional specialization and adaptive evolution of the olfactory system in freshwater fish.

**Abstract:**

Olfaction plays a crucial role in fish feeding behaviors and ecological adaptation. However, systematic studies on its transcriptional regulation and molecular evolutionary mechanisms in herbivorous and carnivorous fishes remain scarce. In this study, we analyzed four Xenocyprididae species: two herbivorous (*Ctenopharyngodon idella* and *Megalobrama amblycephala*) and two carnivorous (*Elopichthys bambusa* and *Culter alburnus*), using olfactory rosette transcriptome sequencing and cross-species comparisons. The number of unigenes per species ranged from 40,229 to 42,405, with BUSCO completeness exceeding 89.2%. Functional annotation was performed using six major databases. Olfactory-related candidate genes were identified based on Pfam domains (7tm_4) and KEGG pathways (ko04740), revealing 8–19 olfactory receptor genes per species. These candidate genes were predominantly enriched in the olfactory transduction and neuroactive ligand–receptor interaction pathways. A total of 3681 single-copy orthologous genes were identified, and their expression profiles exhibited clear interspecific divergence without forming strict clustering by dietary type. High-threshold differentially expressed trend genes (|log_2_FC| ≥ 4) were enriched in pathways related to RNA processing, metabolite transport, and xenobiotic metabolism, suggesting that the olfactory system may participate in diverse adaptive responses. Ka/Ks analysis indicated that most homologous genes were under purifying selection, with only 0.87–2.07% showing positive selection. These positively selected genes were enriched in pathways related to immune response and neural regulation, implying potential roles in adaptive evolution associated with ecological behavior. Furthermore, the olfactory-related gene *oard1* exhibited Ka/Ks > 1 in the *E. bambusa* vs. *C. idella* comparison. qRT-PCR validation confirmed the reliability of the RNA-Seq data. This work is the first to integrate two complementary indicators—expression trends and evolutionary rates—to systematically investigate the transcriptional regulation and molecular evolution of the olfactory system in Xenocyprididae species under the context of dietary differentiation, providing valuable reference data for understanding the perceptual basis of dietary adaptation in freshwater fish.

## 1. Introduction

Olfaction is the primary sensory system by which fish detect chemical signals in their aquatic environment and plays a crucial role in behaviors such as foraging, reproduction, migration, population recognition, and predator avoidance [1]. Compared to other senses such as vision and hearing, olfaction offers several advantages, including longer perception duration, greater detection range, and high sensitivity to various water-soluble compounds, especially in turbid or low-visibility environments [2,3,4]. Fish olfactory recognition primarily relies on the olfactory rosette, where olfactory sensory neurons express multiple receptor gene families that play a crucial role in detecting chemical cues, including main olfactory receptors (MORs), class A G protein–coupled receptors (ORAs), class C receptors (OlfCs), trace amine–associated receptors (TAARs), and formyl peptide receptors (FPRs). Collectively, these gene families form a highly diverse and specialized olfactory system [5,6,7,8]. Numerous studies have shown that the morphological structure of the olfactory rosette, the types and expression patterns of olfactory receptor genes vary significantly among different fish species and are closely associated with their ecological niches, feeding habits, and environmental adaptations [9,10,11,12,13,14].

Fish feeding habits can be categorized as herbivory, carnivory, omnivory, and filter feeding based on dietary preferences and food selection under natural or aquaculture conditions. From a broad perspective, factors such as species genetic background, developmental stage, environmental conditions, food availability, and artificial feeding interventions can all influence the feeding habits of fish [15,16,17]. At a more specific level, feeding behavior is influenced not only by physiological factors such as digestive tract structure and digestive enzyme activity, but also by their sensory systems, particularly the olfactory system, which plays a crucial role in food selection and feeding behavior [18,19,20]. Research has shown that olfactory receptor genes in fish with different dietary habits exhibit significant differences in gene family composition and expression patterns [12,21,22]. For instance, carnivorous species tend to express receptors that detect amino acids and peptides, whereas herbivorous species preferentially express receptors associated with plant-derived compounds [23,24]. Although the essential role of olfaction in feeding regulation has been preliminarily demonstrated, systematic investigations into the expression profiles, functional differentiation, and evolutionary dynamics of olfactory genes in fish with contrasting diets remain limited. In particular, it remains unclear whether herbivorous and carnivorous species exhibit consistent expression trends or signals of positive selection in their olfactory systems.

The family Xenocyprididae, which belongs to the class Actinopteri and order Cypriniformes, represents a taxonomically diverse and ecologically adaptable group of freshwater fish in China. These species are predominantly distributed in the freshwater basins of the East Asian plains, with virtually no natural populations found upstream of the Tiger Leaping Gorge in the Jinsha River or the Hukou Waterfall in the Yellow River [25,26]. Some species have also been introduced to western China, Europe, and North America. At present, the family comprises approximately 45 genera and 160 recognized species, exhibiting highly diversified feeding strategies that range from filter-feeding and omnivory to specialized herbivory and carnivory [27,28,29]. Due to their relatively close phylogenetic relationships yet markedly distinct dietary preferences, Xenocyprididae serve as an ideal model for investigating the adaptive mechanisms and regulatory processes underlying feeding behavior in fish. Previous studies have mainly addressed their phylogeny, morphological divergence, and the evolution of feeding-related structures, revealing preliminary associations between dietary traits and morphological features such as mouth morphology and gill raker configuration [30,31]. However, within this family, the transcriptional regulation and molecular evolutionary patterns of the olfactory system in the context of divergent feeding habits remain insufficiently characterized, leaving it unclear whether dietary divergence is reflected at the molecular level of olfactory regulation and adaptation. Clarifying these mechanisms is not only important for understanding sensory adaptation and evolutionary processes in fishes, but may also provide useful insights for aquaculture, such as feed optimization and husbandry practices, and for conservation efforts by emphasizing the chemosensory requirements that influence habitat use.

Building on this context, the present study focuses on four representative Xenocyprididae species with distinct feeding habits: two herbivorous species (*Ctenopharyngodon idella* and *Megalobrama amblycephala*) and two carnivorous species (*Elopichthys bambusa* and *Culter alburnus*). *C. idella* is widely distributed across major river systems in China and primarily feeds on aquatic macrophytes, while *M. amblycephala* mainly consumes algae and plant detritus. In contrast, *E. bambusa* and *C. alburnus* are typical carnivorous species that prey predominantly on small fish and crustaceans. This study focuses on olfactory organ tissues, aiming to explore the adaptive characteristics of the olfactory system in fish with different feeding habits from two dimensions: transcriptional regulation and molecular evolution. To this end, we generated RNA-seq datasets derived from olfactory rosettes and performed de novo transcriptome assemblies, followed by systematic cross-species comparisons. Specifically, the study (1) identified olfactory-related candidate genes based on transcriptome annotation and analyzed their functional composition and pathway enrichment profiles; (2) performed clustering and trend analysis of expression profiles based on single-copy orthologous genes to uncover feeding-related transcriptional regulatory patterns; and (3) identified genes showing significant expression trends and positively selected genes based on log_2_FC and Ka/Ks ratios, respectively, and examined their potential roles in ecological adaptation through enrichment analysis. This work may provide new molecular insights and a theoretical basis for understanding the olfactory mechanisms underlying dietary differentiation in freshwater fish.

## 2. Materials and Methods

### 2.1. Sample Collection and Olfactory Rosette Processing

Samples were collected in January 2024 from four Xenocyprididae species inhabiting the Jingjiang section of the Yangtze River, China (31.95428° N, 120.12725° E). These included the carnivorous *E. bambusa* and *C. alburnus*, and the herbivorous *C. idella* and *M. amblycephala*. For each species, six healthy adult individuals were selected. The average total length and body mass of the sampled individuals were: *C. idella* (585.17 ± 13.12 mm, 3563.50 ± 63.97 g), *M. amblycephala* (297.83 ± 5.78 mm, 545.05 ± 33.65 g), *C. alburnus* (447.83 ± 18.19 mm, 906.95 ± 12.39 g), and *E. bambusa* (582.83 ± 13.39 mm, 2377.12 ± 28.96 g). Age and gonadal stage were not assessed; however, all specimens were confirmed as adults based on body size and external morphology, which minimized potential variation associated with developmental or reproductive status. Following the administration of MS-222 (120 mg/L) anesthesia for 90 s, the olfactory rosettes were rapidly dissected. The tissues were rinsed with phosphate-buffered saline (PBS), transferred to RNA stabilization solution, and flash-frozen in liquid nitrogen. In order to concentrate on interspecific expression trends and reduce individual variation, the olfactory rosettes from six individuals of each species were pooled in equal amounts for RNA extraction and sequencing. All animal experiments were approved by the Ethics Committee of Shanghai Ocean University (Approval No. SHOU-DW-2023-208) and conducted in accordance with relevant animal use regulations.

### 2.2. RNA Extraction, Library Construction, and Sequencing

Total RNA was extracted using TRIzol reagent (Invitrogen, Waltham, MA, USA), and its concentration and purity were assessed with a NanoDrop 2000 spectrophotometer (Thermo Fisher Scientific, Waltham, MA, USA). The integrity of the RNA was evaluated by agarose gel electrophoresis, and the RNA Quality Number (RQN) was determined using an Agilent 5300 Fragment Analyzer (Agilent Technologies, Santa Clara, CA, USA). The mRNA was enriched using Oligo(dT)-attached magnetic beads and fragmented into approximately 300 base pair fragments with a fragmentation buffer. First-strand cDNA was synthesized using random primers and reverse transcriptase, followed by second-strand synthesis to generate double-stranded cDNA. The termini of the cDNA fragments were then subjected to a repair process that involved the addition of an A-tail. Subsequently, Y-shaped adapters were ligated to the fragments. The ligated products were then purified, size-selected, and amplified by PCR to obtain the final libraries. The high-throughput sequencing was performed on the Illumina NovaSeq 6000 platform (Illumina Inc., San Diego, CA, USA).

### 2.3. De Novo Transcriptome Assembly and Quality Assessment

Raw sequencing reads were subjected to quality control using fastp (https://github.com/OpenGene/fastp (accessed on 11 September 2025)) to remove adapter sequences, low-quality reads, reads with high N content, and overly short sequences, resulting in high-quality clean data. To preserve species-specific transcriptional information, Trinity (https://github.com/trinityrnaseq/trinityrnaseq/wiki (accessed on 11 September 2025)) was employed to perform de novo assembly independently for each species using their respective clean data sets. The initial assembly results were optimized using TransRate (http://hibberdlab.com/transrate/ (accessed on 11 September 2025)), and redundant transcripts were removed using CD-HIT (http://weizhongli-lab.org/cd-hit/ (accessed on 11 September 2025)) to generate a non-redundant set of transcripts. Assembly completeness was assessed with BUSCO (http://busco.ezlab.org (accessed on 11 September 2025)) based on the presence of conserved single-copy orthologous genes. In addition, N50 values and transcript length distributions were calculated to evaluate assembly quality. Finally, the clean reads from each sample were mapped back to the assembled reference transcripts to obtain alignment statistics. The Illumina sequence data generated during the current study are accessible through BioProject accession number PRJNA1163501.

### 2.4. Functional Annotation of Unigenes

Functional annotation was conducted on the non-redundant unigenes assembled for each species. Initially, open reading frames (ORFs) were predicted using TransDecoder (https://github.com/TransDecoder/TransDecoder (accessed on 11 September 2025)). The resulting sequences were then aligned against six major databases: NR, Swiss-Prot, Pfam, EggNOG, GO, and KEGG. The annotation coverage for each database was calculated concurrently. Protein functions were assigned based on sequence similarity using Diamond and Blast+ against the NR and Swiss-Prot databases. EggNOG was used to obtain COG classifications and orthologous group (OG) information via Diamond, while GO terms were assigned and categorized into biological process (BP), cellular component (CC), and molecular function (MF). KEGG pathways were identified through ID mapping, and conserved protein domains were detected by aligning sequences to the Pfam database using HMMER.

### 2.5. Identification and Functional Characterization of Olfaction-Related Candidate Genes

To identify candidate functional genes related to olfaction, two annotation strategies were employed in this study: (1) unigenes annotated to the KEGG olfactory transduction pathway (ko04740), and (2) unigenes containing the typical olfactory receptor domain (PF13853, 7tm_4) as identified in the Pfam database. For each species, gene sets derived from both annotations were merged and subjected to clustering and de-redundancy using CD-HIT with a similarity threshold of 80%, resulting in a non-redundant set of olfaction-related candidate genes.

To further understand the functional composition of olfactory-related candidate genes, multi-gene set enrichment analysis was performed based on the union of the two gene sets before redundancy removal. KEGG enrichment analysis was performed using an R script (Fisher’s exact test, Benjamini–Hochberg correction, *p*-adjust < 0.05), and the results were visualized using a bubble chart. This analysis aimed to elucidate the functional composition characteristics and biological background of olfactory receptor-related genes. The enrichment statistics only reflect functional distribution trends and are not used for statistical inference.

### 2.6. Clustering and Phylogenetic Tree Analysis Based on Single-Copy Orthologous Gene Expression Profiles

To compare the expression regulation characteristics of conserved genes in different Xenocyprididae species, we conducted an analysis based on single-copy orthologous. First, we used OrthoFinder (https://github.com/davidemms/OrthoFinder (accessed on 11 September 2025)) to align the protein sequences of the four species and identify single-copy orthologous gene groups (Orthogroups). Next, we used RSEM (http://deweylab.biostat.wisc.edu/rsem (accessed on 11 September 2025)) in combination with bowtie2 to align the clean reads of each species to their own assembled transcripts, counted the read counts at the gene level, and converted them to TPM values to represent expression levels.

For each single-copy orthologous gene identified, extract the TPM values of the corresponding annotated CDS unigenes in each species to construct a species × gene expression matrix. To eliminate differences in gene expression levels and focus on relative expression trends, standardize the matrix using Z-scores. The standardized expression matrix is used for: (1) bidirectional clustering analysis: Using the R package pheatmap (v1.0.13), hierarchical clustering is performed on both genes (rows) and species (columns) using Euclidean distance and average linkage, and the overall distribution of expression patterns is visualized as a heatmap. (2) expression profile phylogenetic tree construction: Based on the Euclidean distance between species in the standardized expression matrix, the SciPy library (v1.16.0, linkage and dendrogram functions) in Python (v3.11.9) was used to construct a species expression profile phylogenetic tree using the UPGMA method.

### 2.7. Analysis of Differential Expression Trends and Functional Enrichment Based on Single-Copy Orthologous Genes

Since this study employed a pooled-sample design (six individuals per species mixed together) for cross-species expression comparison, traditional differential expression statistical tests are not applicable. To investigate the trends in expression regulation differences among different feeding habits of Xenocyprididae, we used the MARS method (MA-plot-based method with random sampling) from DEGSeq to analyze four species pair combinations (control vs. experimental: *C. idella* vs. *C. alburnus*, *C. idella* vs. *E. bambusa*, *M. amblycephala* vs. *C. alburnus*, *M. amblycephala* vs. *E. bambusa*).

The screening of differentially expressed trend genes (DETGs) follows three criteria: (1) The gene set is limited to single-copy orthologous genes identified by OrthoFinder (excluding paralogous interference); (2) An empirical threshold of |log_2_(Fold Change)| ≥ 4 is used to define strong expression change trends; (3) *p*-values and false discovery rates (FDR) generated by MARS are used solely as references for gene ranking and not as criteria for statistical significance. To explore the potential biological functions of DETGs, functional enrichment analysis was conducted using the full set of annotated single-copy orthologous genes as the background. GO enrichment analysis was performed using Goatools (Fisher’s exact test, Benjamini–Hochberg correction, *p*-adjust < 0.05), while KEGG pathway enrichment was conducted following the same method as described in Section 2.5.

### 2.8. Analysis of Positive Selection Detection and Adaptive Gene Functional Enrichment

To detect selection pressures associated with dietary differentiation, this study employed the same herbivorous-carnivorous species pairing design as in the expression trend analysis (experimental vs. control: *C. alburnus* vs. *C. idella*, *C. alburnus* vs. *M. amblycephala*, *E. bambusa* vs. *C. idella*, *E. bambusa* vs. *M. amblycephala*), and comparative analysis was conducted based on the single-copy orthologous gene CDS sequences of each paired combination.

First, multiple sequence alignments were performed using PRANK, followed by calculation of the non-synonymous substitution rate (Ka) and synonymous substitution rate (Ks) using KaKs Calculator, resulting in the Ka/Ks ratios for each gene. Significance testing was conducted using Fisher’s exact test to control for false positives. Genes with Ka/Ks > 1 were classified as positively selected genes, those with Ka/Ks < 0.1 as highly conserved genes under purifying selection, and the remaining genes (neutral/weakly selected) were excluded from further analysis. GO/KEGG enrichment analysis was performed separately on the selected positively selected genes and conserved genes (methods as in Section 2.7). By independently analyzing the enrichment results of the four paired combinations (taking the top 20 entries sorted by *p*-adjust), functional terms that occurred in ≥3 combinations were selected as the core functional set related to adaptive evolution.

### 2.9. qRT-PCR Validation

To validate the reliability of RNA-seq data, qRT-PCR was performed to verify gene expression levels. Seven representative unigenes were selected based on the olfactory transduction pathway. *β-actin* was used as the internal reference gene, and the relative expression levels of target genes were calculated using the 2^−ΔΔCt^ method [32]. Primer design was performed using Primer Premier 6 software (Table S1), and primers were synthesized by Meiji Biotechnology Co., Ltd. (Shanghai, China). qRT-PCR reactions were conducted on an ABI 7300 quantitative real-time PCR instrument (ABI, New York, NY, USA), with three biological replicates per sample. For downstream analyses, both qRT-PCR relative expression values (2^−ΔΔCt^) and RNA-seq expression values (TPM) were transformed to log_2_(Expression + 1). For bar chart visualization, mean ± SD was calculated from the log_2_-transformed qRT-PCR replicates. To account for gene-specific differences in expression scales, both RNA-seq and qRT-PCR values were further subjected to z-score transformation within genes before correlation analysis between the two platforms.

Unless otherwise specified, all analyses in this study were performed using the default parameters of the respective tools.

## 3. Results

### 3.1. Transcriptome Data Statistics

RNA quality was assessed prior to sequencing. Except for *M. amblycephala*, which exhibited a slightly lower RNA quality number (RQN) of 7.4, all other samples had RQN values above 8.5, indicating high RNA integrity. Subsequent transcriptomic sequencing using the Illumina NovaSeq 6000 platform yielded high-quality clean data, with output ranging from 6.35 to 7.17 Gb and Q30 base percentages exceeding 95.5% across all samples (Table S2).

**Figure 1:**
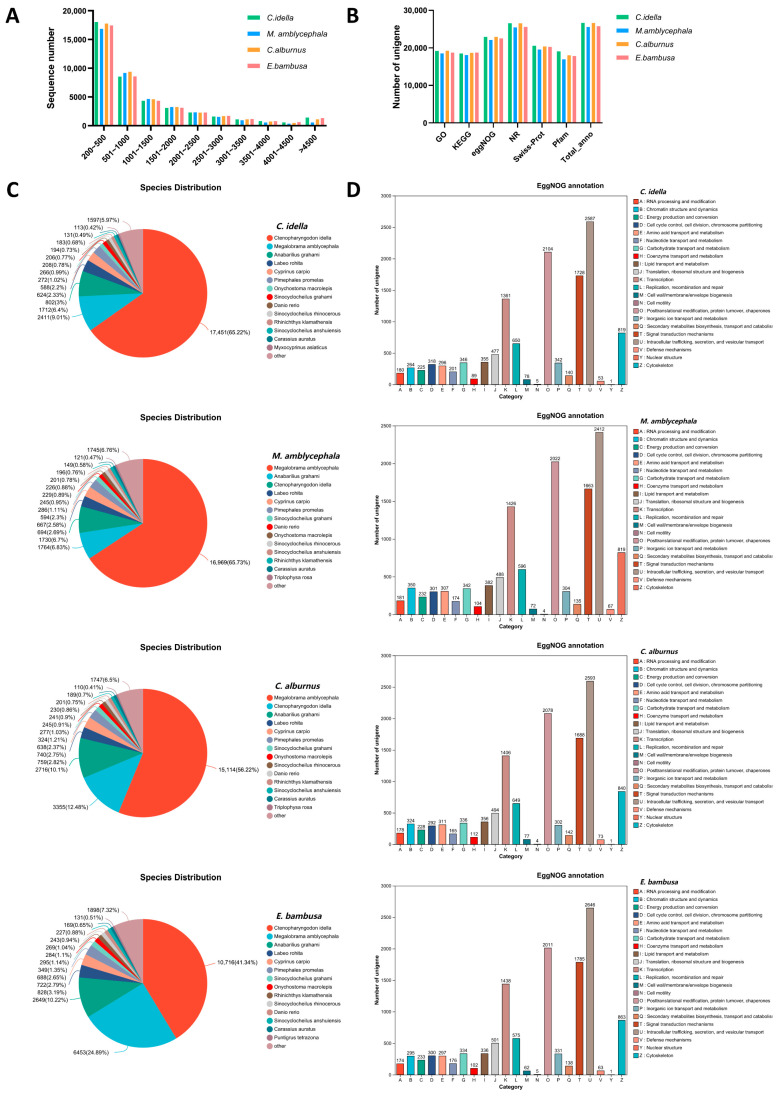
Statistics of unigene length, cross-database annotations, and functional categorization. Note: (**A**) Length distribution of unigenes. The x-axis shows unigene length ranges; the y-axis shows the number of unigenes within each range. (**B**) Bar chart of functional annotations across multiple databases. The x-axis denotes database names; the y-axis denotes the number of sequences annotated in each database. (**C**) Pie chart of species distribution based on NR annotations; colors indicate matched species and proportions. (**D**) Bar chart of EggNOG functional categories. The x-axis shows EggNOG categories (capital letters A–Z); the y-axis shows the number of unigenes assigned to each category.

All clean data were de novo assembled using Trinity, and the assembly results were optimized and summarized (Table S3). The number of unigenes obtained for per species ranged from 40,229 to 42,405, with N50 values ranging from 1805 bp to 2217 bp. TransRate scores exceeded 0.46 for all assemblies. BUSCO analysis indicated high completeness, with scores above 92.7% for all species except *M. amblycephala* (89.2%). Statistical analysis of unigene lengths revealed that 99% of unigenes in all samples were longer than 200 bp, with approximately 90% concentrated between 200 and 3000 bp (Figure 1A). When clean reads were aligned to the Trinity-assembled transcript reference sequences, the alignment rates were all above 79.74%. Finally, based on Salmon quantitative analysis, the number of expressed unigenes (>0 TPM) was 41,289 (*C. idella*), 39,385 (*M. amblycephala*), 41,492 (*C. alburnus*), and 40,646 (*E. bambusa*), respectively.

### 3.2. Functional Annotation of Unigenes

Functional annotation of unigenes from the four fish species (*C. idella*, *M. amblycephala*, *C. alburnus*, and *E. bambusa*) was conducted using six major databases, including NR, Swiss-Prot, Pfam, EggNOG, GO, and KEGG (Table S4, Figure 1B). The number of unigenes annotated in at least one database was 26,672 for *C. idella*, 25,576 for *M. amblycephala*, 26,653 for *C. alburnus*, and 25,785 for *E. bambusa*. The distribution patterns of functional categories in EggNOG, GO, and KEGG were highly consistent across species.

Most unigenes of each species were annotated to the NR database and showed the highest homology with protein sequences of fish species such as *C. idella*, *M. amblycephala*, *Anabarilius grahami*, *Labeo rohita*, and *Cyprinus carpio* (Figure 1C). Functional classification of orthologous groups (OGs) based on EggNOG revealed conserved functional profiles among species (Figure 1D). The five most prevalent functional categories in all species were: Intracellular trafficking, secretion, and vesicular transport [U]; Posttranslational modification, protein turnover, chaperones [O]; Signal transduction mechanisms [T]; Transcription [K]; and Cytoskeleton [Z].

The annotation patterns of the four species are highly consistent across the three major categories of the Gene Ontology (GO) (Figure S1A). In the Molecular Function (MF) category, the items with the most annotated genes are binding (GO:0005488) and catalytic activity (GO:0003824); in Cellular Component (CC), they are cell part (GO:0044464) and membrane (GO:0016020); in Biological Process (BP), they are cellular process (GO:0009987) and metabolic process (GO:0008152). Unigenes were annotated to KEGG pathways to elucidate their involvement in biological processes (Figure S1B). All species were annotated to six major metabolic pathways: environmental information processing (EIP), human diseases (HD), organismal systems (OS), cellular processes (CP), genetic information processing (GIP), and metabolism (M). The top six pathway categories with the highest number of annotated unigenes for each species were concentrated in signal transduction (EIP), cancer: overview (HD), infectious disease: viral (HD), immune system (OS), transport and catabolism (CP), and endocrine system (OS) pathways. Notably, pathways related to chemosensory and neural regulation, such as olfactory transduction (ko04740) and neuroactive ligand-receptor interaction (ko04080), and taste transduction (ko04742), as well as key metabolic and signaling pathways influencing feeding behavior, such as AMPK signaling pathway (ko04152) and insulin signaling pathway (ko04910), were annotated to varying numbers of unigenes. The widespread annotation of these key pathways suggests their potential roles in environmental sensing (particularly food recognition), feeding behavior regulation, and energy metabolism in Xenocyprididae species.

### 3.3. Identification and Functional Characterization of Olfactory-Related Candidate Genes

To identify candidate genes associated with olfactory function, a dual-annotation strategy was employed, combining Pfam domain (7tm_4, PF13853) and KEGG pathway (ko04740) information to screen unigenes from the four Xenocyprididae species. The results showed that a total of 64 (*C. idella*), 74 (*M. amblycephala*), 76 (*C. alburnus*), and 88 (*E. bambusa*) candidate unigenes were identified; based on Pfam annotation; based on KEGG annotation, 101 (*C. idella*), 122 (*M. amblycephala*), 118 (*C. alburnus*), and 122 (*E. bambusa*) candidate unigenes were identified, respectively.

The two types of gene sets identified for each species were merged, and CD-HIT clustering with an 80% similarity threshold was applied to remove redundancy, yielding a non-redundant combined set of domain- and pathway-based candidate genes. To account for clustering units containing multiple unigenes with identical annotations but varying expression levels, the unigene with the highest expression level in each cluster was retained to construct the final functionally non-redundant olfactory candidate gene set (Table S5). The number of genes was 39 (*C. idella*), 36 (*M. amblycephala*), 46 (*C. alburnus*), and 48 (*E. bambusa*), respectively. Among these, the number of genes annotated as olfactory receptor genes was 8 (*C. idella*), 15 (*M. amblycephala*), 17 (*C. alburnus*), and 19 (*E. bambusa*), respectively.

To explore the potential functions of these candidate genes, we further conducted KEGG pathway enrichment analysis using the union of domain-type and pathway-type unigenes before redundancy removal (Figure S2). The top 20 enrichment results showed that the candidate genes of the four species were enriched in the olfactory transduction pathway, supporting their functional relevance to olfaction. Additionally, they were widely enriched in a series of pathways related to neural signal transduction and behavioral regulation, including neuroactive ligand-receptor interaction, circadian entrainment, cAMP signaling pathway, calcium signaling pathway, dopaminergic synapse, adrenergic signaling in cardiomyocytes, gastric acid secretion, GnRH signaling pathway, and oxytocin signaling pathway.

Furthermore, there are certain differences in the distribution of the top 20 enriched pathways among species. For example, the phototransduction pathway is enriched in *C. alburnus*, *E. bambusa*, and *M. amblycephala* but not detected in *C. idella*, suggesting that some species may exhibit synergistic regulation of olfactory and visual signals. The Salivary secretion and long-term potentiation pathways co-occur in *C. idella* and *E. bambusa*, which may be associated with olfactory learning and odor memory. The glutamatergic synapse and insulin secretion pathways are only observed in *M. amblycephala*, suggesting that olfactory candidate genes may participate in neuro-metabolic coupling processes. The renin secretion pathway is only present in *C. alburnus*, potentially reflecting its specialized function in regulating homeostasis.

### 3.4. Identification and Expression Analysis of Orthologous Genes

A total of 3681 single-copy orthologous genes were identified across the transcriptomes of the four species (Table S6). Based on their TPM values, a Z-score-normalized expression matrix was constructed and subsequently used for bidirectional hierarchical clustering and expression profile phylogenetic tree analysis to investigate the similarities and differences in transcriptomic regulatory patterns among species.

**Figure 2:**
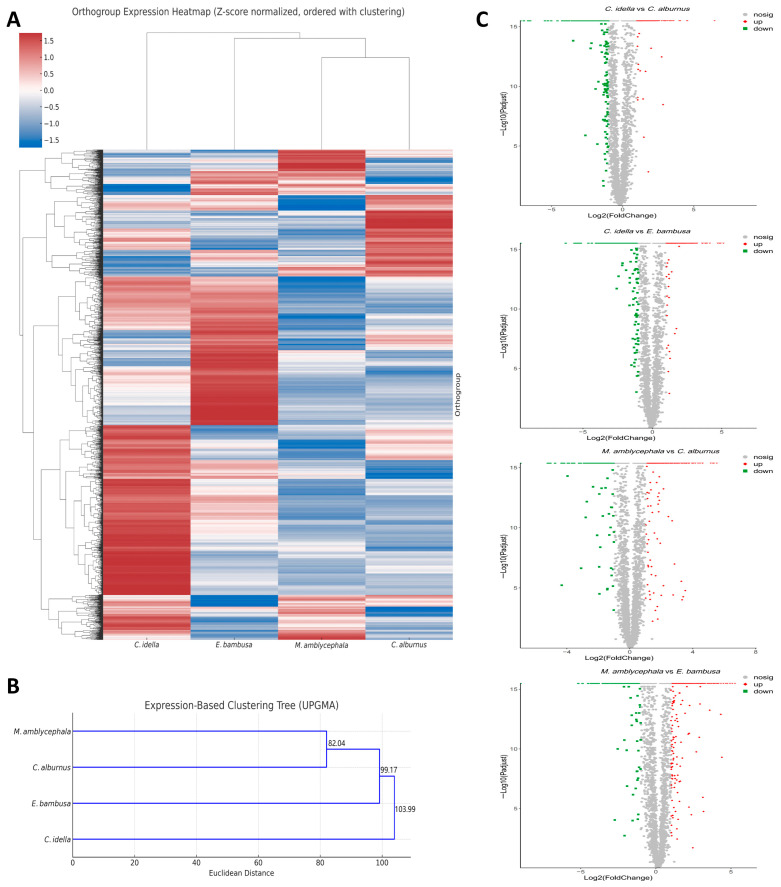
Single-copy-ortholog–driven expression patterns and species clustering. Note: (**A**) Bidirectional hierarchical-clustering heatmap of single-copy orthologs. Rows, orthogroups; columns, species. Dendrograms (left/top) depict clustering; branch lengths reflect expression similarity; colors from blue to red indicate increasing Z-scores. (**B**) Expression-based species clustering tree. The x-axis indicates clustering distance; branch lengths reflect expression dissimilarity; node labels show cluster height. (**C**) Volcano plot based on single-copy orthologs. The x-axis shows fold-change between two sample groups; the y-axis shows *p* values (used for ranking only, not for statistical inference); both axes are log-transformed. Points represent genes: red, up-regulated; green, down-regulated; gray, non-DE.

The bidirectional clustering heatmap (Figure 2A) revealed pronounced expression differences among orthogroups, reflecting a certain degree of species-specific expression trends. Among them, *C. alburnus* and *M. amblycephala* exhibit low expression trends in most orthogroups and cluster into the same branch. In contrast, *C. idella* and *E. bambusa* exhibit relatively high expression levels in most orthogroups, but their orthogroup expression distributions exhibit certain differences, and thus they did not exhibit the same degree of topological consistency as the former in the heatmap. To further analyze the overall similarity among different species at the level of orthologous gene expression profiles, Euclidean distances between species were calculated based on Z-score standardized expression values, and a phylogenetic tree of expression profiles was constructed (Figure 2B). The clustering results showed that the four fish species exhibited a differentiation structure consistent with the heat map on the expression profile phylogenetic tree. *C. alburnus* and *M. amblycephala* first clustered together as a branch with high similarity, and then formed a subclade together with *E. bambusa*. *C. idella* was positioned at a greater clustering distance from this subclade, exhibiting distinct expression patterns that highlight its uniqueness in overall transcriptomic trends.

### 3.5. Analysis of Differential Expression Trends in Orthologous Genes

To explore potential differences in gene expression regulation between herbivorous and carnivorous Xenocyprididae species, we compared expression trends based on DEGSeq analysis for four species pair combinations (*C. idella* vs. *C. alburnus*, *C. idella* vs. *E. bambusa*, *M. amblycephala* vs. *C. alburnus*, and *M. amblycephala* vs. *E. bambusa*). Single-copy orthologous genes showing strong expression differences were defined as DETGs with |log_2_(FoldChange)| ≥ 4. The results showed that the *C. idella* vs. *E. bambusa* pair had the highest number of DETGs (856 in total, with 337 up-regulated and 519 down-regulated), followed by *M. amblycephala* vs. *E. bambusa* (836 in total, with 572 up-regulated and 264 down-regulated), *C. idella* vs. *C. alburnus* (800 in total, with 277 up-regulated and 523 down-regulated) and *M. amblycephala* vs. *C. alburnus* (525 in total, with 357 up-regulated and 168 down-regulated). The distribution of DETGs is presented in a volcano plot (Figure 2C).

### 3.6. Functional Enrichment Analysis of Differentially Expressed Trend Genes

GO and KEGG functional enrichment analyses were performed for the DETGs identified from the four species pair comparisons. For each comparison, the top 60 enriched terms/pathways were extracted for trend assessment.

GO enrichment analysis (Figure S3) revealed that DETGs from multiple species pair comparisons were enriched in functions related to post-transcriptional regulation, predominantly including RNA processing, RNA metabolic process, ribonucleoprotein complex, and RNA binding. These terms were ranked within the top 20 across all four comparisons, indicating a common trend of expression differentiation. These enrichment patterns suggest potential interspecific differences in RNA-level regulatory mechanisms in the olfactory rosette. In addition, terms such as nucleic acid metabolic process and nuclear protein-containing complex were highly enriched in ≥3 comparisons, further supporting the possible core role of nucleic acid metabolism and transcription-associated complexes in gene regulation. By contrast, broader metabolic categories, including cellular macromolecule metabolic process and nucleobase-containing compound metabolic process, were also enriched in multiple groups but ranked relatively lower, and thus can be considered as supplementary trends.

KEGG pathway enrichment analysis (Figure S4) showed that DETGs from the different species pair comparisons were broadly enriched in pathways related to primary metabolism and xenobiotic processing. Metabolic pathways consistently ranked within the top 20 across all four comparisons, indicating a dominant trend of co-enrichment. In addition, pathways such as drug metabolism, metabolism of xenobiotics by cytochrome P450, and focal adhesion were consistently enriched in ≥ 3 comparisons, suggesting interspecific divergence in the metabolic processing of drugs, odorants, and other exogenous compounds in the olfactory rosette, as well as potential divergence in cellular structural organization and signal integration. Notably, chemical carcinogenesis and pathways in cancer also ranked highly in multiple comparisons; in this context, these terms are more likely to reflect the involvement of metabolic enzyme systems or signal transduction modules rather than pathological processes. The biological relevance of these pathways should be further interpreted in light of the specific physiological functions of the olfactory rosette and the ecological niches of the studied species.

### 3.7. Ka/Ks Analysis

Compared with differences in gene expression regulation, selection signals reflect slower and more stable evolutionary process, thereby providing complementary insights into species divergence. To investigate these evolutionary patterns, Ka/Ks analysis was performed for the four species pairs. The results (Table S7) showed that the majority of genes in all pairings were under purifying selection (Ka/Ks < 1, *p* < 0.05), indicating a high degree of functional conservation in core biological processes. The proportion of genes under positive selection (Ka/Ks > 1, *p* < 0.05) was low across all comparisons (0.87–2.07%), but this nevertheless suggests that a subset of genes may have experienced stronger adaptive pressures during species evolution. Notably, the *E. bambusa* vs. *C. idella* comparison exhibited the highest proportion of positively selected genes (2.07%), implying the presence of stronger adaptive evolutionary signals in this pairing, which merits further investigation.

Building on this, we further focused on the evolutionary patterns of olfactory-related candidate genes. By comparing the non-redundant olfactory-related candidate genes from each species with the single-copy orthologous gene dataset, a total of 70 orthogroup (OG) identifiers were matched. Further analysis revealed that the *oard1* gene (OG0009235) exhibited a Ka/Ks ratio greater than 1 in the *E. bambusa* vs. *C. idella* comparison, suggesting that it may potentially be involved in adaptive evolution related to olfactory neural homeostasis or oxidative stress in carnivorous fish species.

### 3.8. Functional Enrichment Analysis of Adaptive Genes

To explore the conservation and adaptive evolutionary characteristics of genes in the olfactory rosette of fish with different feeding habits, Ka/Ks analysis was performed for the four species pair combinations. Genes under conservative evolution (Ka/Ks < 0.1) and those under positive selection (Ka/Ks > 1) were screened separately, followed by GO and KEGG functional enrichment analyses. For each combination, the top 20 enriched terms were retained, and functional terms recurring in at least three combinations were summarized.

**Figure 3:**
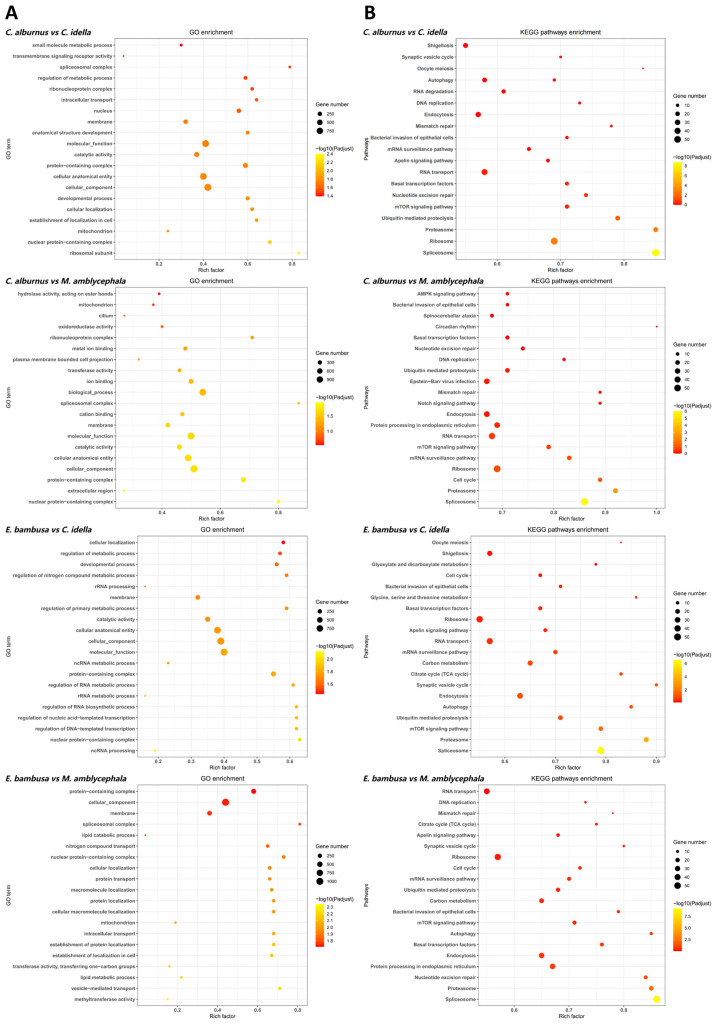
GO and KEGG enrichment analyses (top 20) of conserved genes. Note: (**A**) GO top 20 bubble plot. The y-axis shows GO terms; the x-axis shows the Rich factor. Bubble size indicates the number of genes per term; color encodes ranges of adjusted *p* values (*p*-adjust). (**B**) KEGG top 20 bubble plot. The x-axis shows the Rich factor; the y-axis shows *p*-adjust. Each bubble represents a pathway, with size proportional to the number of enriched unigenes (*p* values are used for ranking only and not for statistical inference).

For conserved genes, GO functional enrichment analysis identified 10 terms that were consistently enriched in ≥3 groups (Figure 3). Among them, membrane, nuclear protein-containing complex, protein-containing complex, and cellular component were enriched across all four groups, mainly related to membrane structure, protein complexes, and fundamental cellular architecture. The other 6 terms, such as catalytic activity and cellular anatomical entity, appeared in three groups. In KEGG pathway analysis, conserved genes were enriched in 17 pathways that were repeatedly enriched in ≥3 comparisons. Among these, 10 pathways were enriched in all four group combinations, including spliceosome, proteasome, ubiquitin-mediated proteolysis, mTOR signaling pathway, basal transcription factors, RNA transport, mRNA surveillance pathway, bacterial invasion of epithelial cells, ribosome, and endocytosis. The remaining 7 pathways, such as DNA replication, mismatch repair, and cell cycle, appeared in 3 groups, with functions primarily related to genetic information processing and cell cycle regulation.

**Figure 4:**
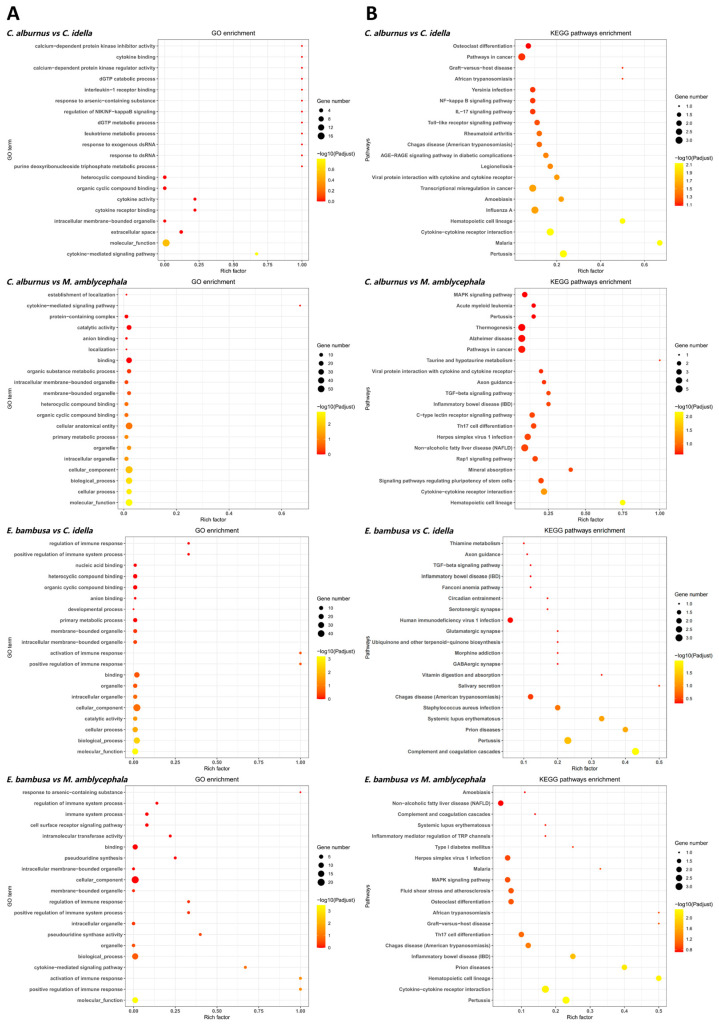
GO and KEGG enrichment analyses (top 20) of positively genes (PSGs). Note: Plot types and symbols as in Figure 3 (*p* values are used for ranking only and not for statistical inference).

For positively selected genes, GO functional enrichment analysis identified 11 terms that were repeatedly enriched in ≥3 combinations (Figure 4), encompassing functions related to organelle structure, signal mediation, and molecular binding. Among these, intracellular membrane-bound organelle and molecular function were enriched across all four combinations, while the remaining nine terms, including cytokine-mediated signaling pathway, heterocyclic compound binding, binding, and organelle, were enriched in three combinations. In the KEGG pathway analysis, five pathways ranked within the top 20 in ≥3 combinations. Of these, the Pertussis pathway was enriched in all four combinations, whereas cytokine–cytokine receptor interaction, hematopoietic cell lineage, inflammatory bowel disease (IBD), and Chagas disease (American trypanosomiasis) were enriched in three combinations. These pathways are mainly associated with cytokine regulation, immune cell development, and inflammatory signaling processes. Notably, the enrichment of IBD specifically in carnivorous fish comparisons may reflect selective pressures related to intestinal immune challenges.

In addition, species-pair-specific patterns were also observed. Some functional terms and pathways appeared in the top 20 of only specific combinations, reflecting inter-combination differences. Among conserved genes, the DNA replication pathway was absent from the top 20 in the *E. bambusa* vs. *C. idella* combination, whereas the cell cycle pathway did not appear in the *C. alburnus* vs. *C. idella* combination. For positively selected genes, the IBD pathway ranked in the top 20 only in combinations involving *M. amblycephala* (*E. bambusa* vs. *M. amblycephala*, *C. alburnus* vs. *M. amblycephala*).

### 3.9. qRT-PCR Validation of Differentially Expressed Trend Genes

**Figure 5:**
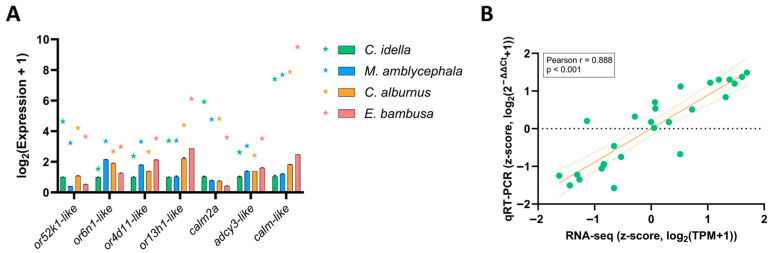
Validation of RNA-seq results by qRT-PCR. Note: (**A**) Comparison of RNA-seq and qRT-PCR expression levels for seven olfactory-related genes across four Xenocyprididae species. Bars represent qRT-PCR values (mean ± SD, n = 3), and stars indicate RNA-seq values; both were transformed to log_2_(Expression+1). Colors denote species: green (*C. idella*), blue (*M. amblycephala*), orange (*C. alburnus*), and red (*E. bambusa*). (**B**) Correlation between RNA-seq and qRT-PCR expression after z-score transformation (28 gene-species data points). Green dots represent individual gene-species data points. The x-axis shows RNA-seq z-scores; the y-axis shows qRT-PCR z-scores. The solid orange line indicates the fitted linear regression, while the dashed lines show the 95% confidence interval. Pearson’s *r* = 0.888, *p* < 0.001.

To verify the reliability of the RNA-seq results, seven representative unigenes from the olfactory transduction pathway were selected for qRT-PCR analysis. Both qRT-PCR relative expression values (2^−ΔΔCt^, mean ± SD, n = 3) and RNA-seq expression levels (TPM) were transformed to log_2_(Expression+1) prior to comparison. As shown in Figure 5A, the qRT-PCR results exhibited expression trends that were generally consistent with those obtained from RNA-seq across the four Xenocyprididae species.

When directly comparing log_2_-transformed values between RNA-seq and qRT-PCR, only a moderate correlation was observed (Pearson *r* = 0.304, *p* < 0.116; Figure S5). This may reflect inherent differences in quantification scales between the two methods. After z-score transformation within genes, however, a strong positive correlation was detected (Pearson *r* = 0.888, *p* < 0.001; Figure 5B). These findings demonstrate that, despite minor numerical differences, qRT-PCR effectively confirmed the relative expression patterns revealed by RNA-seq, thereby supporting the robustness and re-producibility of the transcriptomic data.

## 4. Discussion

This study, through comparative transcriptomic sequencing of the olfactory rosettes from four herbivorous and carnivorous Xenocyprididae species, integrated expression trend analysis with molecular evolutionary signals to systematically uncover adaptive differences in their olfactory systems, providing novel molecular evidence for dietary adaptation in freshwater fish.

### 4.1. Expression Profiling Clustering Reveals Cross-Dietary Expression Similarities and Species-Specific Patterns

Clustering analysis of expression profiles based on 3681 single-copy orthologous genes revealed that, although the four species exhibited distinct differentiation in expression trends, the clustering pattern did not align strictly with herbivorous and carnivorous feeding types. For example, the herbivorous *M. amblycephala* and the carnivorous *C. alburnus* clustered together, whereas the herbivorous *C. idella* displayed relatively independent expression characteristics. These findings suggest that the clustering pattern primarily reflects species-specific regulatory features. The expression profiles of the olfactory system shaped not only by feeding habits but also by phylogenetic history, life-history traits, and habitat-related factors [33].

From an ecological perspective, *C. idella* and *M. amblycephala* predominantly feed on aquatic plants and algae, typically inhabit middle to upper water layers, and rely primarily on visual cues for locating and identifying plant resources. In contrast, *C. alburnus* and *E. bambusa* primarily prey on small, motile organisms, with predation often occurring at dawn, dusk, or in low-light conditions. During long-distance chases, these species are likely to depend more heavily on olfactory detection of diffusive chemical cues. Such differences in sensory reliance may help explain the observed foraging patterns [34]. These interpretations are based on species-level averages from pooled samples, and future studies incorporating repeated behavioral and physiological measurements will be essential for validation.

### 4.2. Olfactory-Related Gene Composition Supports Multi-Pathway Sensory Integration

Joint annotation using Pfam domains (7tm_4) and KEGG pathways (ko04740) revealed that carnivorous species (*E. bambusa*, *C. alburnus*) possess more olfactory receptor genes than herbivorous species (*C. idella*, *M. amblycephala*), consistent with the higher demand for chemical sensory discrimination during active predation. This suggests that olfactory recognition may provide adaptive advantages for predatory strategies. However, electrophysiological evidence indicates that *C. idella* exhibits robust responses to amino acid-based odor stimuli [35], implying that its olfactory perception capacity has not declined. Therefore, the independence observed in its expression profile in this study may more likely reflects ecological differences in the frequency or dependency of olfactory pathway utilization. Future work integrating behavioral experiments would help to elucidate the relative contributions of different sensory systems during foraging.

Dietary shifts in fish have been linked to morphological and physiological changes, such as enhanced intestinal development, increased appetite, altered circadian rhythms, and improved digestive and metabolic functions [36]. In this study, the top 20 KEGG enrichment results for olfactory-related candidate genes indicated that the olfactory transduction pathway was consistently enriched across all species, whereas species-specific functional differences were detected in pathways related to neural signal regulation (e.g., neuroactive ligand–receptor interaction, dopaminergic synapse), metabolic regulation (e.g., insulin secretion, renin secretion), and circadian rhythm (circadian entrainment). Additionally, enrichment of the phototransduction pathway across multiple species suggests that olfactory and visual systems may cooperate in environmental perception. Pathways enriched in specific species, such as glutamatergic synapse and salivary secretion, further imply that the olfactory system may participate in cross-modal sensory integration. Collectively, these findings support the hypothesis that the olfactory system functions as a multi-pathway regulatory platform, coordinating perception, behavior, and physiological processes [37].

Notably, the long-term potentiation (LTP) pathway was identified in the olfactory rosette transcriptomes of *C. idella* and *E. bambusa*. LTP, first discovered in the hippocampus, refers to the sustained enhancement of synaptic strength and is considered a fundamental molecular mechanism underlying learning and memory [38,39,40]. Evidence indicates that LTP also occurs in the olfactory bulb and olfactory cortex, enhancing neural responsiveness to specific odor signals [41,42,43]. The detection of LTP-related genes (e.g., *CaMKII*) in the present study suggests that adaptive learning and memory mechanisms for chemical cues may be present in the olfactory systems of fish, with potential significance for predation, reproduction, and migration behavior. Further targeted neurophysiological and behavioral studies will be necessary to validate these inferences.

### 4.3. DETGs Highlight RNA-Level Post-Transcriptional Regulation and Metabolic Pathway Co-Regulation

The olfactory receptor (OR) gene family exhibits distinct post-transcriptional regulatory features in neurons, enabling rapid responses to olfactory stimuli [44]. In this study, high-threshold differentially expressed trend genes (DETGs) were enriched in multiple species pairs for GO terms such as RNA processing, RNA metabolic process, ribonucleoprotein complex, and RNA binding. These results suggest that the olfactory system in fish may rely extensively on post-transcriptional regulatory mechanisms at the RNA level for rapid adaptation to changes in external chemical cues [45]. Furthermore, the enrichment of the focal adhesion pathway in carnivorous species may be associated with coordinated roles in signal transduction and structural stability within the olfactory epithelium. This pathway is involved in cell adhesion, cytoskeletal remodeling, and signal transduction. Notably, the olfactory epithelium of zebrafish can achieve structural repair and functional recovery after injury, further supporting the close association between olfactory epithelial structure, signal integration, and tissue maintenance [46].

In addition, KEGG pathway analysis further revealed that DETGs were consistently enriched in metabolic regulation pathways across multiple species combinations, including metabolic pathways, drug metabolism, metabolism of xenobiotics by cytochrome P450, and focal adhesion. This is consistent with the conservative adaptive patterns of feeding preferences and metabolic strategies observed in fish reported previously [19,47,48]. Considering the functional characteristics of olfactory rosette tissue, these pathways may not only contribute to energy metabolism and the detoxification of exogenous substances but also participate in olfactory receptor signal transduction and clearance mechanisms. This suggests a potential trend toward coordinated regulation between the olfactory and metabolic systems in maintaining sensory sensitivity and ecological adaptation. Overall, our results indicate that the olfactory system of carnivorous Xenocyprididae species differs from that of herbivorous species not only in sensory function but also in exhibiting specific advantages in RNA-level and metabolic regulation, thereby supporting its adaptive capacity to respond to environmental changes.

### 4.4. Molecular Evolutionary Signals Indicate Adaptive Selection in Immune-Related Pathways

The nasal mucosa of vertebrates can initiate localized adaptive immune responses upon invasion by parasites or pathogens. By employing region-specific immune strategies, it strengthens immune defense while preserving sensory function, supporting the hypothesis that the olfactory system may also act as an “immune sentinel” [49,50]. In this study, Ka/Ks analysis detected a proportion of positively selected genes (0.87–2.07%) in carnivorous fish species, with pronounced enrichment in immune-related pathways, including cytokine–cytokine receptor interaction, hematopoietic cell lineage, and inflammatory bowel disease. These results suggest that the olfactory system may have co-evolved with mucosal immune mechanisms to enhance the detection of food-associated pathogens and environmental toxins [51]. Furthermore, carnivorous fish species exhibited comparatively greater enrichment of immune pathways than herbivorous fish species, possibly reflecting the higher microbial diversity and antigenic load associated with predatory feeding strategies. Such ecological pressures may have driven the co-evolution of the olfactory system and mucosal immunity, thereby improving environmental sensing and defense capabilities [52].

### 4.5. Study Limitations and Future Directions

This study employed a mixed-sample strategy to construct cross-species transcriptomic datasets, which improved the comparability of expression trends but inevitably reduced the statistical power of conventional differential expression analyses [53]. To mitigate this limitation, we combined high fold-change filtering with DEGseq-based trend analysis and validated expression changes in key genes using qRT-PCR, thereby increasing the robustness of our conclusions. Nevertheless, the expression trends reported here should be interpreted as species-level regulatory characteristics, rather than as direct indicators of expression uniformity among individuals.

Previous systematic analyses in model systems such as *Drosophila* have shown that genome-based strategies can achieve higher accuracy and detect a greater quantity of genes than de novo assemblies, when well-annotated reference genomes are available [54]. At the time our experiments were initiated, however, the genome assembly of *E. bambusa* had not yet been released, which limited the feasibility of genome-based approaches. Although genome assemblies have recently been released for all four examined species, *E. bambusa* still lacks official annotation files (e.g., GTF/GFF, CDS, or protein datasets), precluding direct extraction of a consistent gene set from its genome [55]. Accordingly, we applied a de novo transcriptome assembly strategy for the olfactory rosette to obtain comparable sets of expressed transcripts under uniform procedures across all species. Similar approaches have been widely used in cross-species transcriptomic studies of non-model organisms, as they help reduce potential bias from incomplete references and directly capture expressed transcripts [56,57]. Through this strategy, we established a cross-species transcriptomic resource that complements existing genome assemblies. Future integration with newly annotated genomes will further improve gene identification and enhance evolutionary inference.

Integrating the results of expression trend analysis with molecular evolutionary signals, the olfactory system of Xenocyprididae species appears to follow an evolutionary pattern of “overall stability with localized plasticity” in response to dietary divergence. This pattern may represent a key mechanism enabling freshwater fishes to exploit diverse ecological niches [58]. Future studies could build on the present findings by: (1) incorporating biological replicates to strengthen the statistical reliability of differential expression analyses; (2) applying in situ hybridization (ISH) or immunohistochemistry (IHC) to determine the tissue-level localization of key genes; (3) integrating olfactory behavioral assays with gut microbiome profiling to explore functional links between gene expression and dietary adaptation; and (4) including non-Xenocyprididae outgroups in expression clustering analyses to construct a rooted phylogenetic framework, thereby allowing more precise inference of the evolutionary trajectories and mechanisms underlying olfactory expression regulation.

## 5. Conclusions

This study systematically compared the olfactory rosette transcriptomes of four Xenocyprididae species representing herbivorous and carnivorous feeding types. Through transcriptome assembly and functional annotation, olfactory-related candidate genes were identified, and expression trend analyses of single-copy orthologous genes revealed both conserved expression patterns and divergent regulatory features between feeding types. Functional enrichment analyses indicated that these differences were mainly associated with olfactory transduction, metabolism, and neural regulation pathways.

Further Ka/Ks analysis identified several positively selected genes related to sensory perception, immune processes, and metabolic functions, suggesting adaptive divergence of olfactory systems between herbivorous and carnivorous species during evolution. Overall, these findings provide new insights for understanding the molecular mechanisms underlying olfactory adaptation in Xenocyprididae and lay a solid foundation for future studies in freshwater fish sensory ecology and functional genomics.

## Data Availability

The original contributions presented in the study are included in the article, and further inquiries can be directed to the corresponding author.

## Supplementary Materials

The following supporting information can be downloaded at: https://www.mdpi.com/article/10.3390/ani15182741/s1, Table S1: Sequence of the primer; Table S2: Sequencing data statistics table; Table S3: Assembly result evaluation and transcript length; Table S4: Database annotation statistics table; Table S5: Non-redundant set of olfactory candidate genes; Table S6: Single-copy orthologous gene table; Table S7: Ka/Ks ratio information for species pairing combinations. Figure S1: GO/KEGG functional annotations of unigenes; Figure S2: KEGG functional enrichment of olfactory-related candidate genes; Figure S3: GO enrichment analysis (top 60) of DETGs; Figure S4: KEGG enrichment analysis (top 60) of DETGs; Figure S5: Correlation between RNA-seq and qRT-PCR expression values (log_2_-transformed).

## Abbreviations

The following abbreviations are used in this manuscript.

MORs	Main olfactory receptors
ORA	Olfactory receptors related to class A GPCRs
OlfC	Olfactory receptors related to class C GPCRs
TAARs	Trace amine-associated receptors
FPRs	Formyl peptide receptors
*C. idella*	*Ctenopharyngodon idella*
*M. amblycephala*	*Megalobrama amblycephala*
*E. bambusa*	*Elopichthys bambusa*
*C. alburnus*	*Culter alburnus*
PBS	Phosphate-buffered saline
RQN	RNA quality number
ORF	Open reading frames
GO	Gene Ontology
KEGG	Kyoto Encyclopedia of Genes and Genomes
BP	Biological process
CC	Cellular component
MF	Molecular function
OG	Orthologous groups
DETGs	Differentially expressed trend genes
FDR	False discovery rates
EIP	Environmental Information Processing
HD	Human Diseases
OS	Organismal Systems
CP	Cellular Processes
GIP	Genetic Information Processing
M	Metabolism

## References

[1] Keller-Costa T., Canário A.V., Hubbard P.C. (2015). Chemical communication in cichlids: A mini-review. Gen. Comp. Endocrinol..

[2] Wei K., Chen C.S., Zhang X.G., Guo H.Y., Li C., Ma L., Guo M.L. (2017). Review on olfactory organ and behaviour in fish. Mar. Fish..

[3] Whitlock K.E., Palominos M.F. (2022). The Olfactory Tract: Basis for Future Evolution in Response to Rapidly Changing Ecological Niches. Front. Neuroanat..

[4] Pintos S., Cavallino L., Yañez A.V., Pandolfi M., Pozzi A.G. (2021). Effects of intraspecific chemical cues on the behaviour of the bloodfin tetra *Aphyocharax anisitsi* (Ostariophysi: Characidae). Behav. Process..

[5] Laberge F., Hara T.J. (2001). Neurobiology of fish olfaction: A review. Brain Res. Rev..

[6] Zhu G.L., Tang W.Q., Liu D. (2015). Research progress of olfactory receptor genes in fishes. J. Fish. China.

[7] Korsching S. (2009). The molecular evolution of teleost olfactory receptor gene families. Results Probl. Cell Differ..

[8] Johnstone K.A., Lubieniecki K.P., Koop B.F., Davidson W.S. (2012). Identification of olfactory receptor genes in Atlantic salmon *Salmo salar*. J. Fish Biol..

[9] Policarpo M., Bemis K.E., Laurenti P., Legendre L., Sandoz J.C., Rétaux S., Casane D. (2022). Coevolution of the olfactory organ and its receptor repertoire in ray-finned fishes. BMC Biol..

[10] Jiang H., Du K., Gan X., Yang L., He S. (2019). Massive Loss of Olfactory Receptors But Not Trace Amine-Associated Receptors in the World’s Deepest-Living Fish (*Pseudoliparis swirei*). Genes.

[11] Wang H., Chen L., Dong C., Chen B., Li B., Li X., Xu P. (2021). Genome-wide identification and characterization of olfactory receptor genes in common carp (*Cyprinus carpio*). Gene.

[12] Liu H., Chen C., Lv M., Liu N., Hu Y., Zhang H., Enbody E.D., Gao Z., Andersson L., Wang W. (2021). A Chromosome-Level Assembly of Blunt Snout Bream (*Megalobrama amblycephala*) Genome Reveals an Expansion of Olfactory Receptor Genes in Freshwater Fish. Mol. Biol. Evol..

[13] Whitlock K.E. (2006). The sense of scents: Olfactory behaviors in the zebrafish. Zebrafish.

[14] Hayden S., Bekaert M., Crider T.A., Mariani S., Murphy W.J., Teeling E.C. (2010). Ecological adaptation determines functional mammalian olfactory subgenomes. Genome Res..

[15] Xu J., Yin J., Xu B., Zhang C., Ji Y., Ren Y., Xue Y. (2025). The Effect of Ontogenetic Dietary Shifts on the Trophic Structure of Fish Communities Based on the Trophic Spectrum. Fishes.

[16] Sánchez-Hernández J., Fernández de Larrea I., de Guzmán I., González J.M., Larrañaga A. (2025). Species-specific drivers explain fish feeding and individual niche variation. J. Fish Biol..

[17] Steinberg C.E.W. (2018). Trophic Diversification and Speciation—‘Your Eating Fuels Evolution’. Aquatic Animal Nutrition.

[18] Hofer R., Schiemer F. (1981). Proteolytic activity in the digestive tract of several species of fish with different feeding habits. Oecologia.

[19] Liu Y., Zhai G., Su J., Gong Y., Yang B., Lu Q., Xi L., Zheng Y., Cao J., Liu H. (2024). The Chinese longsnout catfish genome provides novel insights into the feeding preference and corresponding metabolic strategy of carnivores. Genome Res..

[20] Heraud C., Hirschinger T., Baranek E., Larroquet L., Surget A., Sandres F., Lanuque A., Terrier F., Roy J. (2022). Detection and Modulation of Olfactory Sensing Receptors in Carnivorous Rainbow Trout (*Oncorhynchus mykiss*) Fed from First Feeding with Plant-Based Diet. Int. J. Mol. Sci..

[21] Hirose A., Nakamura G., Nikaido M., Fujise Y., Kato H., Kishida T. (2024). Localized Expression of Olfactory Receptor Genes in the Olfactory Organ of Common Minke Whales. Int. J. Mol. Sci..

[22] Guan S.H., Huang X., Liu N., Wang W.M., Liu H. (2024). Evolution and expression patterns of olfactory receptors β subtype in *Megalobrama amblycephala*. J. Huazhong Agric. Univ..

[23] Hara T.J. (1994). Olfaction and gustation in fish: An overview. Acta Physiol. Scand..

[24] Kasumyan A.O. (2004). The Olfactory System in Fish: Structure, Function, and Role in Behavior. J. Ichthyol..

[25] Fricke R., Eschmeyer W.N., Van der Laan R. Eschmeyer’s Catalog of Fishes: Genera, Species, References. http://researcharchive.calacademy.org/research/ichthyology/catalog/fishcatmain.asp.

[26] Li S.Z. (2001). Review on “Fauna Sinica, Osteichthyes, Cypriniformes” II. Sci. Press, 1998. Zool. Syst..

[27] Tang K.L., Agnew M.K., Hirt M.V., Sado T., Schneider L.M., Freyhof J., Sulaiman Z., Swartz E., Vidthayanon C., Miya M. (2010). Systematics of the subfamily Danioninae (Teleostei: Cypriniformes: Cyprinidae). Mol. Phylogenet. Evol..

[28] Tang K.L., Lumbantobing D.N., Mayden R.L. (2013). The Phylogenetic Placement of Oxygaster van Hasselt, 1823 (Teleostei: Cypriniformes: Cyprinidae) and the Taxonomic Status of the Family-Group Name Oxygastrinae Bleeker, 1860. Ichthyol. Herpetol..

[29] Tang K.L., Agnew M.K., Hirt M.V., Lumbantobing D.N., Raley M.E., Sado T., Teoh V.H., Yang L., Bart H.L., Harris P.M. (2013). Limits and phylogenetic relationships of East Asian fishes in the subfamily Oxygastrinae (Teleostei: Cypriniformes: Cyprinidae). Zootaxa.

[30] Kou C.N., Li J., Chen W.T., Gao S., Wu Z., Liu Y.Q. (2023). Comparative study of the morphological variation in the feeding organs of seven Cyprinid species in the lower reaches of the Pearl River. J. Fish. Sci. China.

[31] Liang J., Xiong Z.H., Ma D.Q., Yan W.C., He C.L., Liu H., Xiong S. (2024). Digestive Tract Structure and Feeding Habits in *Gymnocypris Potanini firmispinatus*. Fish. Sci..

[32] Livak K.J., Schmittgen T.D. (2001). Analysis of relative gene expression data using real-time quantitative PCR and the 2^−ΔΔCT^ method. Methods.

[33] Niimura Y., Nei M. (2005). Evolutionary dynamics of olfactory receptor genes in fishes and tetrapods. Proc. Natl. Acad. Sci. USA.

[34] Santacà M., Dadda M., Bisazza A. (2021). The role of visual and olfactory cues in social decisions of guppies and zebrafish. Anim. Behav..

[35] Wildhaber M.L., West B.M., Ditter K.K., Peterson A.S., Calfee R.D., Beaman Z.D. (2023). Herbivorous Grass Carp (*Ctenopharyngodon idella*) Exhibit Greater Olfactory Response to Amino Acids Than Filter-Feeding Bighead (*Hypophthalmichthys nobilis*) and Silver Carp (*Hypophthalmichthys molitrix*). Fishes.

[36] He S., Liang X.F., Li L., Sun J., Wen Z.Y., Cheng X.Y., Li A.X., Cai W.J., He Y.H., Wang Y.P. (2015). Transcriptome analysis of food habit transition from carnivory to herbivory in a typical vertebrate herbivore, grass carp *Ctenopharyngodon idella*. BMC Genom..

[37] Fahad Raza M., Anwar M., Husain A., Rizwan M., Li Z., Nie H., Hlaváč P., Ali M.A., Rady A., Su S. (2022). Differential gene expression analysis following olfactory learning in honeybee (*Apis mellifera* L.). PLoS ONE.

[38] Lømo T. (2003). The discovery of long-term potentiation. Philos. Trans. R. Soc. Lond. Ser. B Biol. Sci..

[39] Cooke S.F., Bliss T.V. (2006). Plasticity in the human central nervous system. Brain.

[40] Bliss T.V., Collingridge G.L. (1993). A synaptic model of memory: Long-term potentiation in the hippocampus. Nature.

[41] Wilson D.A., Best A.R., Sullivan R.M. (2004). Plasticity in the olfactory system: Lessons for the neurobiology of memory. Neuroscientist.

[42] Ennis M., Linster C., Aroniadou-Anderjaska V., Ciombor K., Shipley M.T. (1998). Glutamate and synaptic plasticity at mammalian primary olfactory synapses. Ann. N. Y. Acad. Sci..

[43] Fletcher M.L., Wilson D.A. (2003). Olfactory bulb mitral-tufted cell plasticity: Odorant-specific tuning reflects previous odorant exposure. J. Neurosci..

[44] Shum E.Y., Espinoza J.L., Ramaiah M., Wilkinson M.F. (2015). Identification of novel post-transcriptional features in olfactory receptor family mRNAs. Nucleic Acids Res..

[45] Di Liegro C.M., Schiera G., Schirò G., Di Liegro I. (2024). Role of Post-Transcriptional Regulation in Learning and Memory in Mammals. Genes.

[46] Calvo-Ochoa E., Byrd-Jacobs C.A. (2019). The Olfactory System of Zebrafish as a Model for the Study of Neurotoxicity and Injury: Implications for Neuroplasticity and Disease. Int. J. Mol. Sci..

[47] Ling G., Gu J., Genter M.B., Zhuo X., Ding X. (2004). Regulation of cytochrome P450 gene expression in the olfactory mucosa. Chem. Biol. Interact..

[48] Fatsini E., Bautista R., Manchado M., Duncan N.J. (2016). Transcriptomic profiles of the upper olfactory rosette in cultured and wild Senegalese sole (*Solea senegalensis*) males. Comp. Biochem. Physiol. Part D Genom. Proteom..

[49] Yu Y.Y., Kong W., Yin Y.X., Dong F., Huang Z.Y., Yin G.M., Dong S., Salinas I., Zhang Y.A., Xu Z. (2018). Mucosal immunoglobulins protect the olfactory organ of teleost fish against parasitic infection. PLoS Pathog..

[50] Sepahi A., Casadei E., Tacchi L., Muñoz P., LaPatra S.E., Salinas I. (2016). Tissue Microenvironments in the Nasal Epithelium of Rainbow Trout (*Oncorhynchus mykiss*) Define Two Distinct CD8α+ Cell Populations and Establish Regional Immunity. J. Immunol..

[51] Monod G., Saucier D., Perdu-Durand E., Diallo M., Cravedi J.P., Astic L. (1994). Biotransformation enzyme activities in the olfactory organ of rainbow trout (*Oncorhynchus mykiss*). Immunocytochemical localization of cytochrome P4501A1 and its induction by β-naphthoflavone. Fish. Physiol. Biochem..

[52] Watelet J.B., Strolin-Benedetti M., Whomsley R. (2009). Defence mechanisms of olfactory neuro-epithelium: Mucosa regeneration, metabolising enzymes and transporters. B-ENT.

[53] Liao X., Cheng L., Xu P., Lu G., Wachholtz M., Sun X., Chen S. (2013). Transcriptome analysis of crucian carp (*Carassius auratus*), an important aquaculture and hypoxia-tolerant species. PLoS ONE.

[54] Ockendon N.F., O’Connell L.A., Bush S.J., Monzón-Sandoval J., Barnes H., Székely T., Hofmann H.A., Dorus S., Urrutia A.O. (2016). Optimization of next-generation sequencing transcriptome annotation for species lacking sequenced genomes. Mol. Ecol. Resour..

[55] Li S., Xiong X., Qiu S., Shen Z., He Y., Gao Z., Wan S. (2024). Chromosome-level genome assembly of the yellow-cheek carp *Elopichthys bambusa*. Sci. Data.

[56] Carruthers M., Yurchenko A.A., Augley J.J., Adams C.E., Herzyk P., Elmer K.R. (2018). *De novo* transcriptome assembly, annotation and comparison of four ecological and evolutionary model salmonid fish species. BMC Genom..

[57] Da-Anoy J., Posadas N., Conaco C. (2024). Interspecies differences in the transcriptome response of corals to acute heat stress. PeerJ.

[58] Mantica F., Iñiguez L.P., Marquez Y., Permanyer J., Torres-Mendez A., Cruz J., Franch-Marro X., Tulenko F., Burguera D., Bertrand S. (2024). Evolution of tissue-specific expression of ancestral genes across vertebrates and insects. Nat. Ecol. Evol..

