# Supplementary material for: Comparative Transcriptomics of Olfactory Rosettes Reveals Expression Divergence and Adaptive Evolution in Herbivorous and Carnivorous Xenocyprididae Fishes
Source: Animals (Basel). 2025 Sep 19;15(18):2741. doi: 10.3390/ani15182741 (PMC12466535; doi:10.3390/ani15182741)
Supplement: Supplementary file 1 [file animals-15-02741-s001.zip › Supplementary material/Figure S1-5/Supplementary Figure Legends.pdf]

Figure S1 GO/KEGG functional annotations of unigenes.

Note: (A) GO annotation. The y-axis lists level-2 GO terms; the x-axis shows the number of unigenes; colors indicate the three GO domains (BP/CC/MF). (B) KEGG annotation. The y-axis lists pathway names; the x-axis shows the number of unigenes; colors indicate KEGG classes.

Figure S2 KEGG functional enrichment of olfactory-related candidate genes.

Note: Multi-gene set bubble chart displayed on separate axes. The y-axis shows pathway names; the x-axis shows the Rich factor; bubble size indicates the number of genes in each pathway; color encodes *p*-value ranges.

Figure S3 GO enrichment analysis (top 60) of DETGs.

Note: Each bar represents a GO term. The x-axis shows GO term names and classes (classes indicated in the upper-left legend); the y-axis shows the enrichment ratio (Rich factor). Bar color encodes *p*-value, with darker colors indicating higher significance. Significance marks: *** for $p < 0.001$, ** for $p < 0.01$, * for $p < 0.05$. The right-hand color scale indicates the magnitude of the *p*-value (*p*-values are used for ranking only and not for statistical inference).

Figure S4 KEGG enrichment analysis (top 60) of DETGs.

Note: Each bar represents a KEGG pathway. The x-axis shows pathway names and classes (classes indicated in the upper-left legend); the y-axis shows the enrichment ratio (Rich factor). Bar color encodes *p*-value, with darker colors indicating higher significance. Significance marks: *** for $p < 0.001$, ** for $p < 0.01$, * for $p < 0.05$. The right-hand color scale indicates the magnitude of the *p*-value (*p*-values are used for ranking only and not for statistical inference).

Figure S5 Correlation between RNA-seq and qRT-PCR expression values (log_2-transformed).

Note: Scatter plot of RNA-seq log_2(TPM+1) (x-axis) versus qRT-PCR $log_2(2^{\wedge-\Delta\Delta Ct}+1)$ (y-axis; mean of three replicates). Each dot represents the expression of one gene in one species (7 genes × 4 species = 28 data points). The orange line shows the linear regression with 95% CI (dotted). Pearson's $r = 0.304$, $p < 0.116$.
